# Supplementary material for: Children born after assisted reproduction more commonly carry a mitochondrial genotype associating with low birthweight
Source: Nat Commun. 2024 Feb 9;15:1232. doi: 10.1038/s41467-024-45446-1 (PMC10858059; doi:10.1038/s41467-024-45446-1)
Supplement: Supplementary file 1 — Supplementary Information [file 41467_2024_45446_MOESM1_ESM.pdf]

SUPPLEMENTARY DATA TO FIGURE 1 3

Supplementary Table 1. Prevalence of haplogroups per mode of conception. 3

Supplementary Table 2. Prevalence of subhaplogroups per mode of conception. 4

Supplementary Table 3. Distribution ART and SC individuals with homoplasmic variants outside of the haplogroup. 4

Supplementary Table 4. Distribution of the number of heteroplasmic variants per individual. 5

Supplementary Table 5. Location of the heteroplasmic variants. 5

Supplementary Table 6. Type of heteroplasmic variants. 6

Supplementary Table 7. Mean sum of heteroplasmic loads of the individuals categorized according to their location. 6

SUPPLEMENTARY DATA TO FIGURE 2 7

Supplementary Table 8. (Sub)haplogroup distribution in ART and SC children distributed whether their birth weight was categorized under or above the 10th percentile. 7

Supplementary Table 9. (Sub)haplogroup distribution in ART and SC children distributed whether their birthweight was categorized under or above the 25th percentile. 8

Supplementary Table 10. Distribution of ART and SC individuals with homoplasmic variants outside of the haplogroup distributed whether their birthweight was categorized under or above the 10th percentile. 9

Supplementary Table 11. Distribution of ART and SC individuals with homoplasmic variants outside of the haplogroup distributed whether their birthweight was categorized under or above the 25th percentile. 9

Supplementary Table 12. Distribution of SC individuals with heteroplasmic variants distributed whether their birthweight was categorized under or above the 10th percentile. 10

Supplementary Table 13. Distribution of SC individuals with heteroplasmic variants distributed whether their birthweight was categorized under or above the 25th percentile. 10

Supplementary Table 14. Distribution of ART individuals with heteroplasmic variants distributed whether their birthweight was categorized under or above the 10th percentile. 11

Supplementary Table 15. Distribution of ART individuals with heteroplasmic variants distributed whether their birthweight was categorized under or above the 25th percentile. 11

SUPPLEMENTARY DATA TO FIGURE 3 12

Supplementary Table 16. (Sub)haplogroup distribution in ART individuals exposed to Vitrolife® culture medium and SC children distributed whether their birthweight was categorized under or above the 10th percentile. 12

Supplementary Table 17. (Sub)haplogroup distribution in ART individuals exposed to Vitrolife® culture medium and SC children distributed whether their birthweight was categorized under or above the 25th percentile. 13

Supplementary Table 18. Distribution in ART individuals exposed to Vitrolife® culture medium and SC children with homoplasmic variants outside of the haplogroup distributed whether their birthweight was categorized under or above the 10th percentile. 14

Supplementary Table 19. Distribution in ART individuals exposed to Vitrolife® culture medium and SC children with homoplasmic variants outside of the haplogroup distributed whether their birthweight was categorized under or above the 25th percentile. 14

Supplementary Table 20. Distribution in ART individuals exposed to Vitrolife® culture medium and SC children with heteroplasmic variants distributed whether their birthweight was categorized under or above the 10th percentile 15

Supplementary Table 21. Distribution in ART individuals exposed to Vitrolife® culture medium and SC children with heteroplasmic variants distributed whether their birthweight was categorized under or above the 25th percentile 15

SUPPLEMENTARY DATA TO TABLES 2, 3 AND 4 16

Supplementary Table 22. Binary logistic regression for being born under the 10th birthweight percentile in SC and ART individuals exposed to Vitrolife culture medium. 16

Supplementary Table 23. Binary logistic regression for being born under the 25th birthweight percentile in SC and ART individuals exposed to Vitrolife culture medium. 16

SUPPLEMENTARY DATA TO FIGURE 4 17

Supplementary Table 24. Transmitted variants identified in the ART and SC mother-child pairs. 17

SUPPLEMENTARY DATA TO FIGURE 5 18

Supplementary Table 25. Total number of oocytes retrieved in natural and OS cycles per donor. 18

Supplementary Table 26. Changes in heteroplasmic load after transmission seen in the oocytes of natural and OS cycles from the same 19

Supplementary Table 27. Generalized linear model regressions for exploring the role of maternal ageing and the number of oocytes retrieved on the total and coding mtDNA variants in mother-child pairs and oocytes. 20

SUPPLEMENTARY DATA TO MATERIALS AND METHODS 21

Supplementary Table 28. List of mtDNA positions excluded from the final analysis. 21

Exploratory factor analysis 22

Supplementary Figure 1. 23

Supplementary Table 29. 23

Supplementary Figure 2. 24

Supplementary data to Figure 1

Supplementary Table 1. Prevalence of haplogroups per mode of conception.

Only the haplogroups that were present in more than 10 children were further considered in the analysis. Statistics were performed using a two-sided Fisher's exact test. ART N=270, SC N=181. % are calculated as the number of individuals with the haplogroup divided by the total number of individuals in the category ART or SC. ART: assisted reproductive technologies, SC: spontaneously conceived.

Haplogroup	ART N=270	SC N=181	F test
	%(N)		
B	0.4% (1)	0.6% (1)	
C	0.4% (1)	0.0% (0)	
F	0.4% (1)	0.6% (1)	
H	42.6% (115)	43.6% (79)	P=0.847
HV	3.3% (9)	3.9% (7)	P=0.799
I	1.9% (5)	3.3% (6)	P=0.361
J	10.7% (29)	8.3% (15)	P=0.422
K	9.3% (25)	8.8% (16)	P=1.000
L	1.1% (3)	0.6% (1)	
M	1.9% (5)	0.0% (0)	
N	0.0% (0)	1.1% (2)	
R	0.4% (1)	0.6% (1)	
T	8.5% (23)	11.6% (21)	P=0.332
U	15.2% (41)	11.0% (20)	P=0.261
V	1.5% (4)	2.2% (4)	
W	0.4% (1)	2.2% (4)	
X	1.9% (5)	1.7% (3)	
Y	0.4% (1)	0.0% (0)	

Supplementary Table 2. Prevalence of subhaplogroups per mode of conception.
Statistics were performed using a two-sided Fisher's exact test. ART N=270, SC N=181. % are calculated as the number of individuals with the subhaplogroup divided by the total number of individuals in the category ART or SC. ART: assisted reproductive technologies, SC: spontaneously conceived.

Subhaplogroups	ART % (N)	SC % (N)	F test
H1	16.3% (44)	15.5% (28)	P=0.896
H10	3.0% (8)	1.1% (2)	P=0.328
H2	1.5% (4)	3.9% (7)	P=0.126
H3	3.3% (9)	3.9% (7)	P=0.799
H5	3.0% (8)	5.5% (10)	P=0.220
HV0	2.2% (6)	2.2% (4)	P=1.000
J1	7.0% (19)	4.4% (8)	P=0.313
J2	3.7% (10)	3.9% (7)	P=1.000
K1	7.8% (21)	7.7% (14)	P=1.000
T1	2.2% (6)	4.4% (8)	P=0.267
T2	6.3% (17)	7.2% (13)	P=0.705
U4	5.2% (14)	0.6% (1)	P=0.007
U5	7.8% (21)	7.7% (14)	P=1.000

Supplementary Table 3. Distribution ART and SC individuals with homoplasmic variants outside of the haplogroup.
% are calculated as the number of individuals with a homoplasmic variant in the given region, divided by the total number of individuals in the category ART or SC. ART N=270, SC N=181. The sum of all the percentages does not add up to 100% because individuals can have multiple homoplasmic variants in the different categories. Synonymous and non-synonymous variants are subcategories of protein-coding variants. Statistics were performed using a two-sided Fisher's exact test. ART: assisted reproductive technologies, SC: spontaneously conceived.

Region	ART % (N)	SC % (N)	F test
HV	27.4% (74)	34.3% (62)	P=0.143
Non-coding	3.3% (9)	1.1% (2)	P=0.212
OHR	17.4% (47)	14.9% (27)	P=0.519
TAS	1.5% (4)	0.6% (1)	P=0.653
Synonymous	44.4% (120)	44.8% (81)	P=1.000
Non-synonymous	27.0% (73)	34.3% (62)	P=0.116
Of which potentially pathogenic	1.1% (3)	2.2% (4)	P=0.446
rRNA	9.3% (25)	5.5% (10)	P=0.156
tRNA	7.4% (20)	5.5% (10)	P=0.564
Of which potentially pathogenic	0.0% (0)	1.7% (3)	P=0.064
Total	77.4% (209)	80.1% (145)	P=0.559

Supplementary Table 4. Distribution of the number of heteroplasmic variants per individual.

The variants have been categorized per type. The Supplementary Table shows how many individuals carry 1, 2, 3, 4, 5 or no heteroplasmic variants per category. Synonymous and non-synonymous variants are subcategories of protein-coding variants. Differences in distribution were tested with a two-sided Pearson's Chi Square Test. ART individuals N=270, SC individuals N=181. ART: assisted reproductive technologies, SC: spontaneously conceived. The region "non-coding" in this table represents variants in the HV, non-coding, OHR and TAS regions.

Region	ART individuals with N variants						SC individuals with N variants						Chi-square
	0	1	2	3	4	5	0	1	2	3	4	5	
Total	107	94	52	11	6	0	69	69	28	8	4	3	P=0.329
Synonymous	229	30	9	2	0	0	153	14	11	3	0	0	P=0.269
Non-Synonymous	215	45	9	1	0	0	155	25	0	1	0	0	P=0.067
rRNA	247	22	1	0	0	0	167	13	1	0	0	0	P=0.896
tRNA	253	17	0	0	0	0	168	13	0	0	0	0	P=0.711
Non-coding	190	67	12	1	0	0	112	60	8	1	0	0	P=0.270

Supplementary Table 5. Location of the heteroplasmic variants.

% are calculated as the number of individuals with a heteroplasmic variant in the given region, divided by the total number of individuals in the category ART or SC. ART N=270, SC N=181. Note that the total sum of the absolute numbers of each category does not add up to the total number of variants found in the ART and SC group because the insertions and deletions (ART: N=7 and SC: N=6) in the protein-coding regions are not subcategorized in the synonymous and non-synonymous categories. Statistics were performed using a two-sided Fisher's exact test. ART: assisted reproductive technologies, SC: spontaneously conceived.

	All individuals % (N)	ART % (N)	SC % (N)	F test
HV	19.1% (82)	17.7% (45)	21.0% (37)	P=0.454
Non-coding	4.9% (21)	4.7% (12)	5.1% (9)	P=1.000
OHR	14.2% (61)	13.4% (34)	15.3% (27)	P=0.577
TAS	1.2% (5)	1.2% (3)	1.1% (2)	P=1.000
Synonymous	18.6% (80)	18.5% (47)	18.8% (33)	P=1.000
Non-Synonymous	24.7% (103)	26.8% (68)	19.9% (35)	P=0.109
Of which potentially pathogenic	5.7% (24)	6.5% (16)	4.7% (8)	P=0.525
rRNA	8.4% (36)	8.7% (22)	8.0% (14)	P=0.861
tRNA	6.7% (29)	6.3% (16)	7.4% (13)	P=0.698
Of which potentially pathogenic	3.1% (13)	4.07% (10)	1.8% (3)	P=0.255
Total	417	247	170	

Supplementary Table 6. Type of heteroplasmic variants.

% are calculated as the number of individuals with a heteroplasmic variant of a specific type, divided by the total number of individuals in the category ART or SC. ART N=270, SC N=181. Statistics were performed using a two-sided Fisher's exact test. ART: assisted reproductive technologies, SC: spontaneously conceived.

	Total % (N)	ART % (N)	SC % (N)	F test
Transition	89.1% (383)	89.4% (227)	88.6% (156)	P=0.875
Transversion	3.5% (15)	3.1% (8)	4.0% (7)	P=0.790
Insertion	5.1% (22)	4.7% (12)	5.7% (10)	P=0.663
Deletion	2.3% (10)	2.8% (7)	1.7% (3)	P=0.537

Supplementary Table 7. Mean sum of heteroplasmic loads of the individuals categorized according to their location.

Synonymous and non-synonymous variants are subcategories of protein-coding variants. Statistics were performed using a two-sided Mann-Whitney U Test. ART: assisted reproductive technologies, SC: spontaneously conceived.

	ART	SC	Student t test
HV	4.5 ± 18.2%	7.0 ± 23.1%	P=0.202
Non-coding	0.9 ± 7.6%	1.1 ± 8.0%	P=0.807
OHR	2.1 ± 11.8%	2.5 ± 11.6%	P=0.712
TAS	0.0 ± 0.6%	0.7 ± 5.8%	P=0.074
Synonymous	2.8 ± 11.5%	2.2 ± 8.8%	P=0.580
Non-synonymous	2.7 ± 9.7%	2.1 ± 7.2%	P=0.491
rRNA	1.6 ± 9.3%	1.2 ± 5.5%	P=0.622
tRNA	0.9 ± 7.3%	0.9 ± 4.5%	P=0.930

Supplementary data to Figure 2

Supplementary Table 8. (Sub)haplogroup distribution in ART and SC children distributed whether their birth weight was categorized under or above the 10th percentile.
% are calculated as the number of individuals with a certain (sub)haplogroup, divided by the total number of individuals in the category <P10 (N=28) and >P10 (N=360). ART: assisted reproductive technologies, SC: spontaneously conceived. Statistics were performed using a two-sided Fischer's exact test and corrected for multiple testing using the Bonferroni method

Haplogroups	<P10<br>% (N)	>P10<br>% (N)	P-value
H	53.6% (15)	42.8% (154)	P=0.323
HV	7.1% (2)	3.3% (12)	P=0.268
I	3.6% (1)	2.2% (8)	P=0.494
J	7.1% (2)	10. 6% (38)	P=0.754
K	10.7% (3)	9.2% (33)	P=0.735
T	0% (0)	10.6% (38)	P=0.094
U	7.1% (2)	12.8% (46)	P=0.555
Subhaplogroups			
H1	17. 9% (5)	15.8% (57)	P=0.789
H10	0% (0)	2.5% (9)	P=1.000
H2	0% (0)	3.1% (11)	P=1.000
H3	3.6% (1)	3.3% (12)	P=1.000
H4	3.6% (1)	1.9% (7)	P=0.454
H5	0% (0)	4.2% (15)	P=0.614
HV0	7.1% (2)	1.7% (6)	P=0.107
J1	3.6% (1)	6.4% (23)	P=1.000
J2	3.6% (1)	4.2% (15)	P=1.000
K1	10.7% (3)	7.5% (27)	P=0.467
T1	0% (0)	3.6% (13)	P=0.611
T2	0% (0)	6.9% (25)	P=0.240
U4	0% (0)	3.1% (11)	P=1.000
U5	7.1% (2)	7.5% (27)	P=1.000

Supplementary Table 9. (Sub)haplogroup distribution in ART and SC children distributed whether their birthweight was categorized under or above the 25th percentile.

% are calculated as the number of individuals with a certain (sub)haplogroup, divided by the total number of individuals in the category <P25 (N=67) and >P25 (N=321). Children with haplogroup T appeared underrepresented in the <P25 category, however, when correcting for multiple testing, this association was not statistically significant (p=0.04, p-values ≤0.006 were considered significant). Statistics were performed using a two-sided Fischer's exact test and corrected for multiple testing using the Bonferroni method. ART: assisted reproductive technologies, SC: spontaneously conceived.

Haplogroups	<P25 % (N)	>P25 % (N)	P-value
H	38.8% (26)	44.5% (143)	P=0.419
HV	6.0% (4)	3.1% (10)	P=0.276
I	4.5% (3)	1.9% (6)	P=0.191
J	14.9% (10)	9.3% (30)	P=0.186
K	9.0% (6)	9.3% (30)	P=1.000
T	3.0% (2)	11.2% (36)	P=0.041
U	14.9% (10)	11.2% (38)	P=0.540
Subhaplogroups			
H1	11.9% (8)	16.8% (54)	P=0.365
H10	3.0% (2)	2.2% (7)	P=0.657
H2	1.5% (1)	3.1% (10)	P=0.698
H3	4.5% (3)	3.1% (10)	P=0.477
H5	3.0% (2)	4.0% (13)	P=1.000
HV0	4.5% (3)	1.6% (5)	P=0.144
J1	9.0% (6)	5.6% (18)	P=0.276
J2	6.0% (4)	3.7% (12)	P=0.495
K1	7.5% (5)	7.8% (25)	P=1.000
T1	0.0% (0)	4.0% (13)	P=0.137
T2	3.0% (2)	7.2% (23)	P=0.279
U4	4.5% (3)	2.5% (8)	P=0.412
U5	4.5% (3)	8.1% (26)	P=0.444

Supplementary Table 10. Distribution of ART and SC individuals with homoplasmic variants outside of the haplogroup distributed whether their birthweight was categorized under or above the 10th percentile.

% are calculated as the number of individuals with a certain homoplasmic variant, divided by the total number of individuals in the category <P10 (N=28) and >P10 (N=360). The percentages do not add up to 100% because individuals can have multiple homoplasmic variants in different categories. tRNA homoplasmies were more frequently found in the <P10 group, however, after correcting for multiple testing, this association was not statistically significant (p=0.04, p-values ≤0.007 were considered significant). Statistics were performed using a two-sided Fischer's exact test and corrected for multiple testing using the Bonferroni method. ART: assisted reproductive technologies, SC: spontaneously conceived.

Region	<P10<br>% (N)	>P10<br>% (N)	F test
HV	28.6% (8)	29.7% (107)	P=1.000
Non-coding	7.1% (2)	3.3% (12)	P=0.268
OHR	17.9% (5)	17.5% (63)	P=1.000
TAS	0.0% (0)	1.4% (5)	P=1.000
Synonymous	53.6% (15)	44.2% (159)	P=0.431
Non-synonymous	25.0% (7)	29.7% (107)	P=0.672
rRNA	0.0% (0)	8.3% (30)	P=0.150
tRNA	17.9% (5)	6.1% (22)	P=0.036
Total	82.1% (23)	78.6% (283)	P=0.812

Supplementary Table 11. Distribution of ART and SC individuals with homoplasmic variants outside of the haplogroup distributed whether their birthweight was categorized under or above the 25th percentile.

% are calculated as the number of individuals with a certain homoplasmic variant, divided by the total number of individuals in the category <P25 (N=67) and >P25 (N=321). The percentages do not add up to 100% because individuals can have multiple homoplasmic variants in different categories. Statistics were performed using a two-sided Fischer's exact test. ART: assisted reproductive technologies, SC: spontaneously conceived.

Region	<P25<br>% (N)	>P25<br>% (N)	F test
HV	22.4% (15)	30.5% (98)	P=0.237
Non-coding	11.9% (8)	15.9% (51)	P=0.387
OHR	4.5% (3)	2.2% (7)	P=0.461
TAS	3.0% (2)	0.6% (2)	P=0.139
Synonymous	41.8% (28)	45.5% (146)	P=0.592
Non-synonymous	26.9% (18)	29.9% (96)	P=0.661
rRNA	4.5% (3)	8.1% (26)	P=0.444
tRNA	10.4% (7)	6.2% (20)	P=0.287

Supplementary Table 12. Distribution of SC individuals with heteroplasmic variants distributed whether their birthweight was categorized under or above the 10th percentile. % are calculated as the number of individuals with a certain heteroplasmic variant, divided by the total number of individuals in the category <P10 (N=9) and >P10 (N=155). The percentages do not add up to 100% because individuals can have multiple heteroplasmic variants in different categories. Statistics were performed using the two-sided Fischer's exact test. SC: spontaneously conceived.

Region	<P10 SC % (N)	>P10 SC % (N)	F test
HV	0.0% (0)	20.0% (31)	P=0.210
Non-coding	0.0% (0)	7.1% (11)	P=1.000
OHR	11.1% (1)	14.2% (22)	P=1.000
TAS	0.0% (0)	1.9% (3)	P=1.000
Synonymous	33.3% (3)	14.8% (23)	P=0.154
Non-synonymous	44.4% (4)	17.4% (27)	P=0.066
rRNA	33.3% (3)	7.7% (12)	P=0.038
Non-synonymous + rRNA	66.6% (6)	25.2% (39)	P=0.014
tRNA	11.1% (1)	7.7% (12)	P=0.534

Supplementary Table 13. Distribution of SC individuals with heteroplasmic variants distributed whether their birthweight was categorized under or above the 25th percentile. % are calculated as the number of individuals with a certain heteroplasmic variant, divided by the total number of individuals in the category <P25 (N=26) and >P25 (N=138). The percentages do not add up to 100% because individuals can have multiple heteroplasmic variants in different categories. Statistics were performed using a two-sided Fischer's exact test. SC: spontaneously conceived.

Region	<P25 SC % (N)	>P25 SC % (N)	F test
HV	11.5% (3)	20.3% (28)	P=0.416
Non-coding	11.5% (3)	5.8% (8)	P=0.383
OHR	3.8% (1)	15.9% (22)	P=0.130
TAS	0.0% (0)	2.2% (3)	P=1.000
Synonymous	23.1% (6)	14.5% (20)	P=0.257
Non-synonymous	34.6% (9)	15.9% (22)	P=0.052
rRNA	23.1% (6)	6.5% (9)	P=0.016
Non-synonymous + rRNA	53.8% (14)	22.5% (31)	P=0.003
tRNA	7.7% (2)	8.0% (11)	P=1.000

Supplementary Table 14. Distribution of ART individuals with heteroplasmic variants distributed whether their birthweight was categorized under or above the 10th percentile. % are calculated as the number of individuals with a certain heteroplasmic variant, divided by the total number of individuals in the category <P10 (N=19) and >P10 (N=205). The percentages do not add up to 100% because individuals can have multiple heteroplasmic variants in different categories. Statistics were performed using a two-sided Fischer's exact. ART: assisted reproductive technologies.

Region	<P10 ART % (N)	>P10 ART % (N)	P-value
HV	21.1% (4)	15.1% (31)	P=0.509
Non-coding	5.3% (1)	4.9% (10)	P=1.000
OHR	21.1% (4)	10.7% (22)	P=0.249
TAS	0% (0)	0.98% (2)	P=1.000
Synonymous	31.6% (6)	14.1% (29)	P=0.089
Non-synonymous	15.8% (3)	24.9% (51)	P=0.575
rRNA	5.3% (1)	10.2% (21)	P=0.702
Non-synonymous + rRNA	21.1% (4)	33.2% (68)	P=0.319
tRNA	0% (0)	7.3% (15)	P=0.623

Supplementary Table 15. Distribution of ART individuals with heteroplasmic variants distributed whether their birthweight was categorized under or above the 25th percentile. % are calculated as the number of individuals with a certain heteroplasmic variant, divided by the total number of individuals in the category <P25 (N=41) and >P25 (N=183). The percentages do not add up to 100% because individuals can have multiple heteroplasmic variants in different categories. Statistics were performed using a two-sided Fischer's exact test. ART: assisted reproductive technologies.

Region	<P25 ART % (N)	>P25 ART % (N)	P-value
HV	12.2% (5)	16.4% (30)	P=0.637
Non-coding	4.9% (2)	4.9% (9)	P=1.000
OHR	22.0% (9)	9.3% (17)	P=0.031
TAS	2.4% (1)	0.5% (1)	P=0.333
Synonymous	19.5% (8)	14.8% (27)	P=0.477
Non-synonymous	24.4% (10)	24.0% (44)	P=1.000
rRNA	4.9% (2)	10.9% (20)	P=0.383
Non-synonymous + rRNA	29.3% (12)	32.8% (60)	P=0.715
tRNA	2.4% (1)	7.7% (14)	P=0.316

Supplementary data to Figure 3

Supplementary Table 16. (Sub)haplogroup distribution in ART individuals exposed to Vitrolife® culture medium and SC children distributed whether their birthweight was categorized under or above the 10th percentile.

Statistics were performed using a two-sided Fischer's exact test and corrected for multiple testing using the Bonferroni method. ART: assisted reproductive technologies.

Haplogroup	<P10_SC_Vitrolife® % (N)	>P10_SC_Vitrolife® % (N)	P-value
HG.H	52.9% (9)	43.4% (115)	P=0.461
HG.HV	5.9% (1)	3.8% (10)	P=0.502
HG.I	5.9% (1)	2.3% (6)	P=0.356
HG.J	5.9% (1)	9.4% (25)	P=1.000
HG.K	17.6% (3)	9.1% (24)	P=0.241
HG.T	0.0% (0)	11.3% (30)	P=0.232
HG.U	5.9% (1)	11.3% (30)	P=0.704
Subhaplogroup			
SUBHG.H1	17.6% (3)	15.5% (41)	P=0.735
SUBHG.H10	0.0% (0)	2.6% (7)	P=1.000
SUBHG.H2	0.0% (0)	3.0% (8)	P=1.000
SUBHG.H3	5.9% (1)	3.4% (9)	P=0.469
SUBHG.H4	5.9% (1)	2.3% (6)	P=0.356
SUBHG.H5	0.0% (0)	4.5% (12)	P=1.000
SUBHG.HV0	5.9% (1)	1.9% (5)	P=0.314
SUBHG.J1	5.9% (1)	6.4% (17)	P=1.000
SUBHG.J2	0.0% (0)	3.0% (8)	P=1.000
SUBHG.K1	17.6% (3)	7.2% (19)	P=0.137
SUBHG.T1	0.0% (0)	3.8% (10)	P=1.000
SUBHG.T2	0.0% (0)	7.5% (20)	P=0.619
SUBHG.U4	0.0% (0)	1.5% (4)	P=1.000
SUBHG.U5	5.9% (1)	7.2% (19)	P=1.000

Supplementary Table 17. (Sub)haplogroup distribution in ART individuals exposed to Vitrolife® culture medium and SC children distributed whether their birthweight was categorized under or above the 25th percentile.

Statistics were performed using a two-sided Fischer's exact test and corrected for multiple testing using the Bonferroni method. ART: assisted reproductive technologies, SC: spontaneously conceived.

Haplogroup	<P25_SC_Vitrolife® % (N)	>P25_SC_Vitrolife® % (N)	P-value
HG.H	37.5% (18)	45.3% (106)	P=0.343
HG.HV	6.3% (3)	3.4% (8)	P=0.406
HG.I	6.3% (3)	1.7% (4)	P=0.098
HG.J	16.7% (8)	7.7% (18)	P=0.059
HG.K	10.4% (5)	9.4% (22)	P=0.790
HG.T	2.1% (1)	12.4% (29)	P=0.038
HG.U	12.5% (6)	10.7% (25)	P=0.800
Subhaplogroup			
SUBHG.H1	10.4% (5)	16.7% (39)	P=0.383
SUBHG.H10	2.1% (1)	2.6% (6)	P=1.000
SUBHG.H2	2.1% (1)	3.0% (7)	P=1.000
SUBHG.H3	6.3% (3)	3.0% (7)	P=0.382
SUBHG.H4	2.1% (1)	2.6% (6)	P=1.000
SUBHG.H5	4.2% (2)	4.3% (10)	P=1.000
SUBHG.HV0	4.2% (2)	1.7% (4)	P=0.271
SUBHG.J1	12.5% (6)	5.1% (12)	P=0.096
SUBHG.J2	4.2% (2)	2.6% (6)	P=0.628
SUBHG.K1	8.3% (4)	7.7% (18)	P=0.775
SUBHG.T1	0.0% (0)	4.3% (10)	P=0.221
SUBHG.T2	2.1% (1)	8.1% (19)	P=0.215
SUBHG.U4	2.1% (1)	1.3% (3)	P=0.528
SUBHG.U5	4.2% (2)	7.7% (18)	P=0.544

Supplementary Table 18. Distribution in ART individuals exposed to Vitrolife® culture medium and SC children with homoplasmic variants outside of the haplogroup distributed whether their birthweight was categorized under or above the 10th percentile.
Statistics were performed using a two-sided Fischer's exact. ART: assisted reproductive technologies, SC: spontaneously conceived.

Region	<P10_SC_Vitrolife® % (N)	>P10_SC_Vitrolife® % (N)	P-value
HV	29.4% (5)	30.2% (80)	P=1.000
OHR	23.5% (4)	16.2% (43)	P=0.498
Non-coding	5.9% (1)	2.3% (6)	P=0.356
TAS	0.0% (0)	1.1% (3)	P=1.000
Synonymous	52.9% (9)	44.5% (118)	P=0.617
Non-Synonymous	35.3% (6)	32.1% (85)	P=0.793
rRNA	0.0% (0)	6.8% (18)	P=0.611
tRNA	29.4% (5)	6.4% (17)	P=0.006

Supplementary Table 19. Distribution in ART individuals exposed to Vitrolife® culture medium and SC children with homoplasmic variants outside of the haplogroup distributed whether their birthweight was categorized under or above the 25th percentile.
Statistics were performed using a two-sided Fischer's exact test and corrected for multiple testing using the Bonferroni method. ART: assisted reproductive technologies, SC: spontaneously conceived.

Region	<P25_SC_Vitrolife® % (N)	>P25_SC_Vitrolife® % (N)	P-value
HV	22.9% (11)	31.6% (74)	P=0.300
OHR	14.6% (7)	17.1% (40)	P=0.832
Non-coding	4.2% (2)	2.1% (5)	P=0.340
TAS	4.2% (2)	0.4% (1)	P=0.076
Synonymous	39.6% (19)	46.1% (108)	P=0.430
Non-synonymous	31.3% (15)	32.5% (76)	P=1.000
rRNA	4.2% (2)	6.8% (16)	P=0.747
tRNA	14.6% (7)	6.4% (15)	P=0.073

Supplementary Table 20. Distribution in ART individuals exposed to Vitrolife® culture medium and SC children with heteroplasmic variants distributed whether their birthweight was categorized under or above the 10th percentile.

Statistics were performed using a two-sided Fischer's exact test. ART: assisted reproductive technologies, SC: spontaneously conceived.

Region	<P10_SC_Vitrolife® % (N)	>P10_SC_Vitrolife® % (N)	P-value
HV	5.9% (1)	16.6% (44)	P=0.326
Non-coding	5.9% (1)	6.4% (17)	P=1.000
OHR	23.5% (4)	11.7% (31)	P=0.243
TAS	0.0% (0)	1.5% (4)	P=1.000
Synonymous	35.3% (6)	14.7% (39)	P=0.037
Non-synonymous	35.3% (6)	22.6% (60)	P=0.242
rRNA	23.5% (4)	9.1% (24)	P=0.075
Non-synonymous + rRNA	52.9% (9)	31.3% (83)	P=0.106
tRNA	5.9% (1)	7.5% (20)	P=1.000

Supplementary Table 21. Distribution in ART individuals exposed to Vitrolife® culture medium and SC children with heteroplasmic variants distributed whether their birthweight was categorized under or above the 25th percentile.

Statistics were performed using a two-sided Fischer's exact test. ART: assisted reproductive technologies, SC: spontaneously conceived.

Region	<P25_SC_Vitrolife® % (N)	>P25_SC_Vitrolife® % (N)	P-value
HV	10.4% (5)	17.1% (40)	P=0.176
Non-coding	10.4% (5)	5.6% (13)	P=0.204
OHR	12.5% (6)	12.4% (29)	P=1.000
TAS	2.1% (1)	1.3% (3)	P=0.528
Synonymous	22.9% (11)	14.5% (34)	P=0.192
Non-synonymous	37.5% (18)	20.5% (48)	P=0.015
RNA	16.7% (8)	8.5% (20)	P=0.109
Non-synonymous + rRNA	52.1% (25)	28.6% (67)	P=0.002
tRNA	6.3% (3)	7.7% (18)	P=1.000

Supplementary data to tables 2, 3 and 4

Supplementary Table 22. Binary logistic regression for being born under the 10th birthweight percentile in SC and ART individuals exposed to Vitrolife culture medium.

Method: Backward conditional, all factors input. Variables that are significant and overlap with the forward model shown in table 2 are underlined.

Variable	Significance	Exp(B)	95% CI Exp(B)	
			Lower	Upper
<u>Smoking</u>	.004	25.684	2.813	234.507
<u>Pregnancy hypertension</u>	.020	72.741	1.940	2728.098
Primiparity	.084	5.481	.795	37.776
Haplogroup H	.126	4.857	.643	36.703
Haplogroup HV	.025	47.812	1.637	1396.398
<u>Homoplasmic variant tRNA</u>	<.001	81.464	7.499	884.968
Has heteroplasmy HV	.997	.000	.000	.
Has heteroplasmy OHR	.060	7.579	.921	62.381
Has heteroplasmy rRNA	.048	8.211	1.021	66.044

Supplementary Table 23. Binary logistic regression for being born under the 25th birthweight percentile in SC and ART individuals exposed to Vitrolife culture medium.

Method: Backward conditional, all factors input. Variables that are significant and overlap with the forward model shown in table 2 are underlined.

Variable	Significance	Exp(B)	95% CI Exp(B)	
			Lower	Upper
<u>Maternal age</u>	.017	1.121	1.020	1.232
Has heteroplasmy HV	.070	.243	.053	1.123
<u>Has heteroplasmy nonsyn rRNA</u>	.032	2.426	1.077	5.464

Supplementary data to figure 4

Supplementary Table 24. Transmitted variants identified in the ART and SC mother-child pairs.

Syn: synonymous, ART: assisted reproductive technologies, SC: spontaneously conceived.

Variant	Locus	Amino-acid change or type	Load Mother	Load Child	Group
m.7348T>C	MT-CO1	V482A	1.47	16.2	ART
m.806C>T	MT-RNR1	rRNA	23.1	25.9	ART
m.11773C>T	MT-ND4	Syn	13.3	20.8	ART
m.3197T>C	MT-RNR2	rRNA	90.7	96.5	ART
m.3480A>G	MT-ND1	Syn	8.95	4.0	ART
m.4561T>C	MT-ND2	V31A	8.6	3.5	ART
m.12732T>C	MT-ND5	Syn	23.4	48.9	ART
m.9287G>A	MT-CO3	Syn	30.1	11.9	ART
m.11950A>G	MT-ND4	Syn	18.2	44.0	ART
m.9439G>A	MT-CO3	G78D	2.3	21.3	SC
m.15016C>T	MT-CYB	Syn	70.9	59.1	SC
m.727T>C	MT-RNR1	rRNA	5.9	13.2	SC
m.16291C>T	HV	HV	37.4	34.4	SC
m.7465DelAC	MT-TS1	tRNA	63.8	31.6	SC
m.14566A>G	MT-ND6	Syn	40.6	69.3	SC
m.14755A>T	MT-CYB	Syn	5.7	3.7	SC
m.3916DelGA	MT-ND1	Syn	1.9	1.5	SC
m.16093T>C	HV_2	HV	85.4	97.1	SC
m.7498G>A	MT-TS1	tRNA	1.7	6.5	SC
m.8705T>C	MT-ATP6	M60T	29.3	40.9	SC

Supplementary data to figure 5

Supplementary Table 25. Total number of oocytes retrieved in natural and OS cycles per donor.

Each donor underwent up to three natural menstrual cycle and one cycle after ovarian stimulation (OS). The oocytes from an OS cycle used in this study were supernumerary to the mature oocytes that were suitable for oocyte donation.

Donor	#Natural cycle oocytes	#OS cycle oocytes
1	3	7
2	2	2
3	1	2
4	1	0
5	2	6
6	2	2
7	3	4
8	1	0
9	1	6
10	1	3
11	1	3
13	3	0
14	1	2
15	1	2
16	3	0
17	3	7
18	3	0
19	3	0
20	3	4
21	3	2
22	3	2
23	0	0
24	1	4
25	1	2
26	1	2
27	0	0
28	0	0
29	1	3

Supplementary Table 26. Changes in heteroplasmic load after transmission seen in the oocytes of natural and OS cycles from the same .

No differences were seen between natural and stimulated oocytes, Chi Square, p=0.265

	Natural cycle oocytes % (N)	OS cycle oocyte % (N)
Same	3.0% (1)	11.9% (5)
Decrease	48.5% (16)	31.0% (13)
Increase	48.5% (16)	47.6% (20)

Supplementary Table 27. Generalized linear model regressions for exploring the role of maternal ageing and the number of oocytes retrieved on the total and coding mtDNA variants in mother-child pairs and oocytes.

Model	(B)	95% Wald C.I.	Significance
Total number of *de novo* variants in mother child pairs and oocytes (N=170)			
Maternal age	0.037	0.024 - 0.050	<0.001
Synonymous *de novo* variants in mother child pairs and oocytes (N=170)			
Maternal age	0.009	-0.018 – 0.037	0.515
Non-Synonymous *de novo* variants in mother child pairs and oocytes (N=170)			
Maternal age	0.010	-0.018 - 0.039	0.470
Non-Synonymous and rRNA *de novo* variants in mother child pairs and oocytes (N=170)	0.025	-0.055 – 0.105	0.535
Maternal age			
de novo synonymous variants in oocytes (N=113)			
Maternal age	0.008	-0.006-0.022	0.244
Oocytes retrieved	0.000	-0.006-0.006	0.922
de novo non-synonymous variants in oocytes (N=113)			
Maternal age	0.001	-0.006-0.017	0.937
Oocytes retrieved	0.004	-0.03-0.011	0.234
de novo rRNA variants in oocytes (N=113)			
Maternal age	0.032	0.00001- 0.064	0.05
Oocytes retrieved	0.017	0.003-0.303	0.016

Supplementary data to Materials and Methods

Supplementary Table 28. List of mtDNA positions excluded from the final analysis.

Most of the excluded variants were variants that recurrently appeared across samples. They were frequently located in a homopolymeric stretch and were likely appearing due to PCR/sequencing artifacts. The first 250 bp of each amplicon were excluded as well since their coverage was significantly higher than the rest of the mtDNA sequence, hereby increasing the risk for calling an inaccurate heteroplasmic load.

Position	Region	Reason
303	MT-HV2; MT-OHR	Recurrent
310-316	MT-HV2; MT-OHR	Recurrent
513-515	Non-coding	Recurrent
528-778	MT-TFH; MT-HSP1; MT-TF; MT-RNR1	First 250bp of forward primer
955	MT-RNR1	Recurrent
1174-1424	MT-RNR1	First 250bp of reverse primer
5042-5292	MT-ND2	First 250bp of forward primer
5539-5789	MT-TW; MT-TA; MT-TN; MT-OLR; MT-TC	First 250bp of reverse primer
6286	MT-CO1	Recurrent
10935	MT-ND4	Recurrent
12148	MT-TH	Recurrent
13762	MT-ND5	Recurrent
15399	MT-CYB	Recurrent
15407	MT-CYB	Recurrent
16180-16199	MT-HV1	Recurrent

Exploratory factor analysis

Exploratory factor analysis is a statistical method used to extract the variability among variables in the form of independent latent variables. We hypothesized that the eight variables generated by adding up the heteroplasmic loads of variants of a given type (all variants in the HV, non-coding, OHR, TAS, protein-coding (synonymous and non-synonymous), rRNA-coding and tRNA-coding regions) could potentially be explained by a lower number of underlying variables, or factors. Hence, we carried out an exploratory factor analysis using these eight variables as input. The number of finally chosen factors in the model is based on the eigenvalue. In our dataset, we could decrease the number of variables to four factors, which represented the majority of variance of the eight variables (Supplementary Figure 1a). The relative contribution of each variable to each factor is reflected in the component matrix (Supplementary Figure 1b). Next, SPSS computes the factor scores per sample, for all four factors. To calculate the factor score for a given sample for a given factor, the sample's standardized score on each variable is multiplied by the corresponding normalized loads of the variable for the given factor in the component matrix, and these products are added up (see example in Supplementary Figure 1c). In the example, the heteroplasmic loads are not normalized for the entire population, as done by SPSS, to facilitate the illustration of the principle of the method. As a result, each sample has a corresponding score for each of the four factors, which reflect its mtDNA variant composition. The Supplementary Table 29 shows the correlation matrix of the different variables during the factor analysis. Supplementary Figure 2 illustrates the relationship between the heteroplasmic loads of the variants in each of the regions and the resulting factor, for all individuals in this study. The figure shows as an example factor 2. Since the loads in the HV, NonCod, TAS and OHR negatively influence the value of factor 2, they have been put in the negative, and the loads in Syn, NonSyn and rRNA in the positive as they positively impact the factor score. The data has been split in quartiles based on their factor 2 score rank.

a

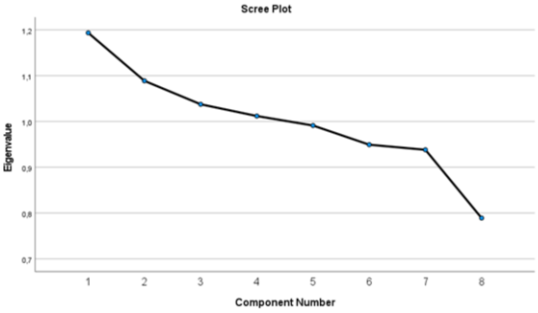

b

Component Matrix	Factors/components 1	2	3	4
HV	0.003	-0.203	-0.131	0.835
Non-Coding	0.052	-0.067	0.737	-0.158
OHR	-0.009	-0.249	-0.194	-0.506
TAS	0.797	-0.230	-0.046	-0.069
Synonymous	0.668	0.356	0.016	0.093
Non-Synonymous	0.121	0.713	0.098	0.099
rRNA	-0.114	0.554	-0.205	-0.104
tRNA	-0.081	-0.001	0.638	0.198

c

Variant	Load	Category
m.11815A>G	32.2%	Syn
m.15616C>T	68.0%	Syn
m.152T>C	6.7%	OHR
m.15119G>A	2.3%	NonSyn

Sum of heteroplasmic loads per variable			
HV	0.0%	Syn	100.2%
NonCod	0.0%	NonSyn	2.3%
OHR	6.7%	rRNA	0.0%
TAS	0.0%	tRNA	0.0%

Score of factor 2

= (0.0 x -0.203) + (0.0 x -0.067) + (0.067 x -0.249) + (0.0 x -0.230) + (1.02 x 0.356) + (0.023 x 0.713) + (0.0 x 0.554) + (0.0 x -0.001)

HV NonCod OHR TAS Syn NonSyn rRNA tRNA

= 0.3628

Supplementary Figure 1.

a Scree plot of the exploratory factor analysis. **b** Component Matrix of the exploratory factor analysis. **c** example of how the factor scores are calculated.

Supplementary Table 29.

Correlation matrix of the different variables in the factor analysis. The variables dominating the scores for each factor are highlighted: Factor 1, Factor 2, Factor 3, Factor 4.

	HV	NonCod	OHR	TAS	Syn	NonSyn	rRNA	tRNA
HV	1.000	-.008	-.043	-.022	.023	-.015	-.019	.025
NonCod	-.008	1.000	-.024	-.011	.015	-.023	-.017	.048
OHR	-.043	-.024	1.000	-.016	-.018	-.046	-.027	-.027
TAS	-.022	-.011	-.016	1.000	.117	-.020	-.003	-.008
Syn	.023	.015	-.018	.117	1.000	.137	.038	-.016
NonSyn	-.015	-.023	-.046	-.020	.137	1.000	.029	.024
rRNA	-.019	-.017	-.027	-.003	.038	.029	1.000	-.026
tRNA	.025	.048	-.027	-.008	-.016	.024	-.026	1.000

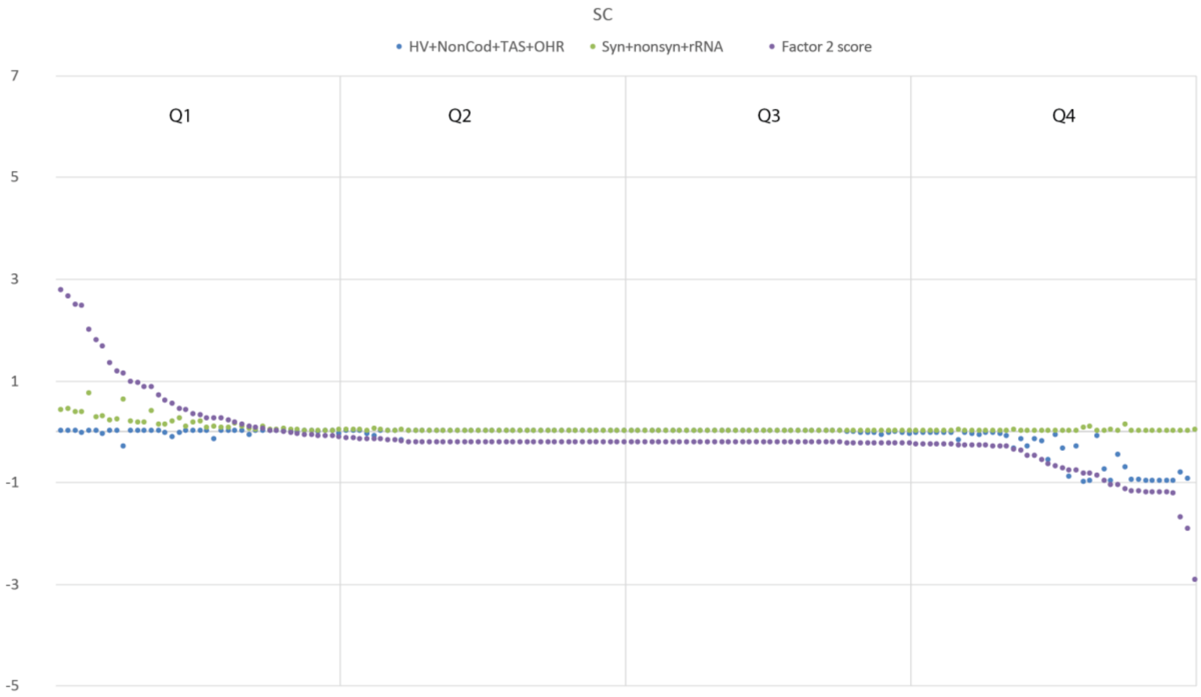

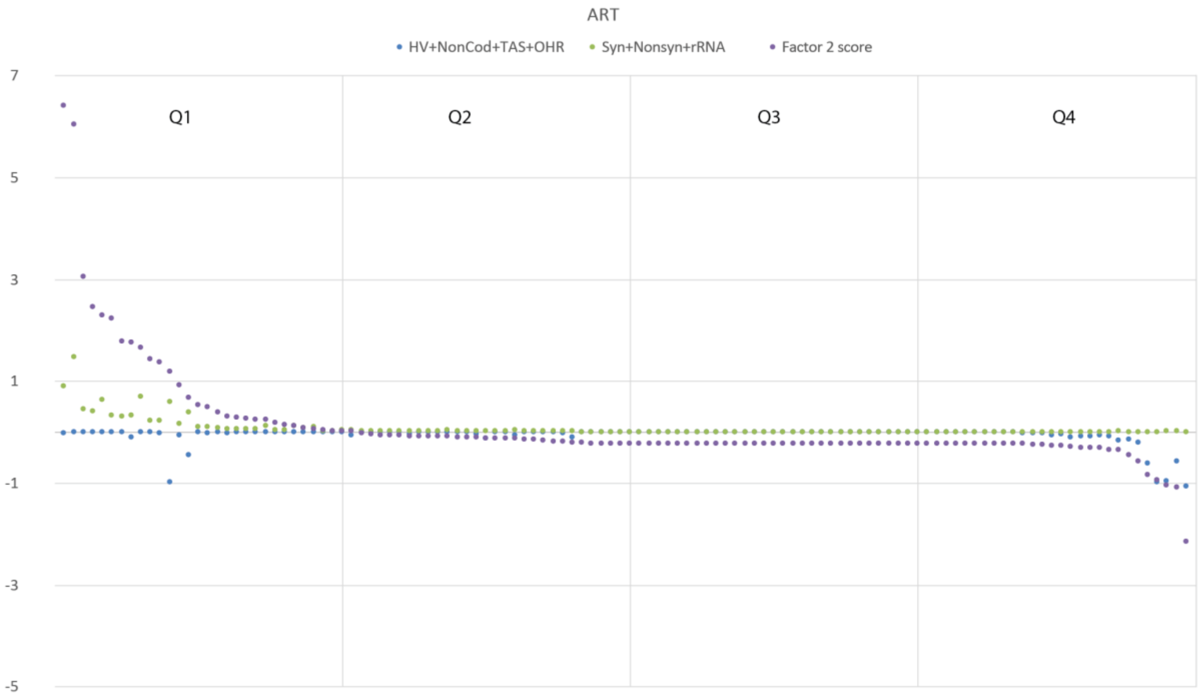

Supplementary Figure 2.

Relationship between the heteroplasmic loads of the variants in each of the regions and the resulting factor 2, for all individuals in this study. Each individual is represented by a triad of dots. The purple dot is their factor 2 score. The blue dot represents the sum of the heteroplasmic loads of the HV, NonCod, TAS and OHR, in per unit (instead of percent). The green dots the sum of loads for syn, nonsyn and rRNA variants.
