# Supplementary material for: Children born after assisted reproduction more commonly carry a mitochondrial genotype associating with low birthweight
Source: Nat Commun. 2024 Feb 9;15:1232. doi: 10.1038/s41467-024-45446-1 (PMC10858059; doi:10.1038/s41467-024-45446-1)
Supplement: Supplementary file 3 — Description of Additional Supplementary Files [file 41467_2024_45446_MOESM3_ESM.pdf]

Description of Additional Supplementary Files

File Name: Supplementary Data 1
Description: Potential pathogenicity of the non-synonymous heteroplasmic variants found in ART individuals exposed to Vitrolife® culture medium and SC children .

File Name: Supplementary Data 2
Description: Univariate analysis of factors versus birthweight percentile in SC individuals and ART exposed to Vitrolife® culture medium. ART: assisted reproductive technologies, SC: spontaneously conceived. Statistical tests were performed with Chi-square test, except for maternal age (indicated as mean ± standard error) which was done with a Mann-Whitney U test. Parameters with a p-value <0.2 were included in further statistical models, except for the homoplasmic variants in TAS in the P25 as they only represent 3 events.

File Name: Supplementary Data 3
Description: De novo variants identified in the ART and SC mother-child pairs. Syn: synonymous, ART: assisted reproductive technologies, SC: spontaneously conceived.

File Name: Supplementary Data 4
Description: Transmitted variants identified in the oocytes. Variants were categorized transmitted when present in at least two samples of the same , irrespective of the origin of the sample (blood, buccal swab (BS), natural cycle (NC) oocytes or ovarian stimulation (OS) oocytes. NonCod: non-coding, Syn; synonymous.

File Name: Supplementary Data 5
Description: De novo variants identified in the oocytes. Variants were considered de novo if they only appeared in one of the oocytes and not in the somatic tissues. NC: natural cycle oocytes, OS: ovarian stimulation oocytes, Syn: synonymous. Empty spaces in the table indicate that there were not as many oocytes.

File Name: Supplementary Data 6
Description: All the variants called in all samples included in this study and clinical parameters used in the study of the birthweight.
