# Supplementary material for: Children born after assisted reproduction more commonly carry a mitochondrial genotype associating with low birthweight
Source: Nat Commun. 2024 Feb 9;15:1232. doi: 10.1038/s41467-024-45446-1 (PMC10858059; doi:10.1038/s41467-024-45446-1)
Supplement: Supplementary file 10 — Reporting Summary [file 41467_2024_45446_MOESM10_ESM.pdf]

Corresponding author(s): Claudia Spits

Last updated by author(s): 18/12/2023

Reporting Summary

Nature Portfolio wishes to improve the reproducibility of the work that we publish. This form provides structure for consistency and transparency in reporting. For further information on Nature Portfolio policies, see our Editorial Policies and the Editorial Policy Checklist.

Statistics

For all statistical analyses, confirm that the following items are present in the figure legend, table legend, main text, or Methods section.

n/a	Confirmed	
☐	☒	The exact sample size (*n*) for each experimental group/condition, given as a discrete number and unit of measurement
☒	☐	A statement on whether measurements were taken from distinct samples or whether the same sample was measured repeatedly
☐	☒	The statistical test(s) used AND whether they are one- or two-sided<br>*Only common tests should be described solely by name; describe more complex techniques in the Methods section.*
☐	☒	A description of all covariates tested
☐	☒	A description of any assumptions or corrections, such as tests of normality and adjustment for multiple comparisons
☐	☒	A full description of the statistical parameters including central tendency (e.g. means) or other basic estimates (e.g. regression coefficient) AND variation (e.g. standard deviation) or associated estimates of uncertainty (e.g. confidence intervals)
☐	☒	For null hypothesis testing, the test statistic (e.g. *F*, *t*, *r*) with confidence intervals, effect sizes, degrees of freedom and *P* value noted<br>*Give P values as exact values whenever suitable.*
☒	☐	For Bayesian analysis, information on the choice of priors and Markov chain Monte Carlo settings
☐	☒	For hierarchical and complex designs, identification of the appropriate level for tests and full reporting of outcomes
☒	☐	Estimates of effect sizes (e.g. Cohen's *d*, Pearson's *r*), indicating how they were calculated

Our web collection on statistics for biologists contains articles on many of the points above.

Software and code

Policy information about availability of computer code

Data collection	Data was collected in Microsoft excel
Data analysis	The full data analysis protocol was published in Mertens et al. 2019 (Bio-Protocol). The software programs used in this study were BWA-MEM, mtDNA Server, Mutect2, MitImpact, MutPred2 and MitoTip. Statistical analysis was done using IBM SPSS Statistics version 28.

For manuscripts utilizing custom algorithms or software that are central to the research but not yet described in published literature, software must be made available to editors and reviewers. We strongly encourage code deposition in a community repository (e.g. GitHub). See the Nature Portfolio guidelines for submitting code & software for further information.

Data

Policy information about availability of data

All manuscripts must include a data availability statement. This statement should provide the following information, where applicable:

- Accession codes, unique identifiers, or web links for publicly available datasets
- A description of any restrictions on data availability
- For clinical datasets or third party data, please ensure that the statement adheres to our policy

Source data for all figures is provided with this paper. The raw sequencing data is considered personal data by the General Data Protection Regulation of the European Union (Regulation (EU) 2016/679) and cannot be publicly shared. The data can be obtained from the corresponding author upon reasonable request and

after signing a Data Use Agreement. All mitochondrial DNA variants called in all samples are provided with this paper in the Supplementary Data 6 file, as well as the clinical parameters associated to the samples.

Research involving human participants, their data, or biological material

Policy information about studies with human participants or human data. See also policy information about sex, gender (identity/presentation), and sexual orientation and race, ethnicity and racism.

Reporting on sex and gender	We only report on sex in this study
Reporting on race, ethnicity, or other socially relevant groupings	We do not report on race, ethnicity or other social groupings
Population characteristics	We provide all relevant recorded details in the manuscript, in table 1. The following co-variates were recorded for the participants: mode of conception, tissue, age, birth weight, gestational age, sex, embryo culture medium, gestational hypertension, gestational diabetes, parity, smoking during pregnancy and body mass index of the mother.
Recruitment	Recruitment is described in the methods section.: The DNA samples extracted from blood from newborns were supernumerary material recruited at the UZ Brussel for a study designed to test if paternal mitochondrial DNA can be found in individuals born after ISCI(Danan et al., 1999). The parents gave informed consent. The ethical commission gave permission to utilize the archived DNA samples for this study, but not the associated clinical parameters. The 9-years old samples were recruited at the MUMC, as part of a follow-up study on a prospective observational cohort study (Dumoulin et al., 2010). The parents were approached after the ninth birthday of their child to participate in this study. The local ethics committee approved the study and both parents of all children gave written informed consent. The DNA was extracted from saliva samples. The placental DNA samples were collected for a study coordinated by the MUMC(Kleijkers et al., 2014, 2016; Mulder et al., 2020a). Ethical approval was requested but was waived, as in accordance with Dutch law, spare placenta tissues can be used for research after informed consent of the patient, without further permission of an ethical committee since no interventions were needed to obtain the samples. The placental biopsies were obtained in six IVF clinics in the Netherlands (Amsterdam UMC, location AMC in Amsterdam, Catharina Hospital in Eindhoven, St. Elisabeth Hospital in Tilburg, Maastricht University Medical Center in Maastricht and University Medical Center Groningen in Groningen) and five IVF clinics affiliated to these hospitals, all patients signed informed consent (Kleijkers et al., 2016). Details on sample collection and extraction can be found in previously published work (Kleijkers et al., 2016; Mulder et al., 2020b). The 18-years old ART individuals, the samples were collected as part of a larger project on the cardio-metabolic and reproductive health of young adult women and men born after ICSI conducted at the UZ Brussel(Belva et al., 2016, 2017). Young adult ICSI men, born between 1992 and 1996 after transfer of fresh ICSI embryos using autologous, freshly ejaculated sperm, are part of a cohort that has been prospectively monitored since birth. All parents of eligible ICSI offspring in our database were sent a letter explaining the background and set-up of the study. Shortly after, these parents were contacted by phone in order to explore their and their children's willingness to participate. After parental consent was obtained, the young adults were approached directly and invited to participate. All participants signed informed consent and received an incentive by means of a gift voucher. The DNA was extracted form peripheral blood. The local ethical committee of the UZ Brussel approved this study. The 18-years old SC individuals were recruited first year students at the campus of the Vrije Universiteit Brussel. They were approached during lecturing, where they received an information brochure on the study, an informed consent form and the necessary materials to provide a buccal swab. The volunteers that enrolled in the study signed the informed consent prior to providing the sample. This study was approved by the local ethical committee of the UZ Brussel.<br>For the study of oocytes, twenty-nine young women donated oocytes in up to three natural menstrual cycles and after one OS cycle. All donors were recruited at the Center for Reproductive Medicine of the UZ Brussel, as part as the standard oocyte donation programme, where also all medical procedures took place. All donors provided informed consent for all medical procedures and for the use of their oocytes for research, and received financial compensation. The local ethics committee of the UZ Brussel approved all parts of this study.<br>We do not identify specific aspects to the recruitment that could have introduce a bias in the study.
Ethics oversight	The local ethical committee of the UZ Brussel and the ethical review board of the Maastricht University Medical Centre. All participants or their parents signed informed consent.

Note that full information on the approval of the study protocol must also be provided in the manuscript.

Field-specific reporting

Please select the one below that is the best fit for your research. If you are not sure, read the appropriate sections before making your selection.

☒ Life sciences ☐ Behavioural & social sciences ☐ Ecological, evolutionary & environmental sciences

For a reference copy of the document with all sections, see nature.com/documents/nr-reporting-summary-flat.pdf

Life sciences study design

All studies must disclose on these points even when the disclosure is negative.

Sample size	When calculating the sample size needed to observe an effect, we first established a pilot study on a smaller set of DNA samples (20 samples of ART and SC individuals). The results showed that three times more individuals in the ART group carried combined loads over 30% in the coding regions than in the control group. With this data, we established that the required sample size, with a power of 0.8 and an alpha of 5%,

	to test if the combined heteroplasmic load is higher in the ART than the control group, is of 202 individuals in each group. We strove to meet this sample size during the sample collection. After curating of the sequencing data, we had a sample size of 270 ART and 181 SC individuals.
Data exclusions	Samples were excluded when they were of non-European origin and when they were of the same family (siblings).
Replication	The sequencing and variant calling method has been previously established in our laboratory during which DNA samples were sequenced multiple times to ensure the robustness of low-frequency variant calling (published in Mertens et al., 2019, Zambelli et al., 2017). To replicate the findings of this study, a new study should be initiated to sequence the mitochondrial genome of another cohort of ART and SC individuals.
Randomization	Samples were not randomized but analysis was carried out considering mode of conception and sex and statistically controlled for other co-variates.
Blinding	Blinding during patient recruitment was not possible for the individuals recruiting them, but the investigators were blinded during sample preparation, the sequencing and variant calling. The link between clinical parameters and mitochondrial DNA genotypes was done only after completion of the all DNA analyses, after which statistical analysis was carried out.

Reporting for specific materials, systems and methods

We require information from authors about some types of materials, experimental systems and methods used in many studies. Here, indicate whether each material, system or method listed is relevant to your study. If you are not sure if a list item applies to your research, read the appropriate section before selecting a response.

Materials & experimental systems

n/a	Involved in the study
☒	☐ Antibodies
☒	☐ Eukaryotic cell lines
☒	☐ Palaeontology and archaeology
☒	☐ Animals and other organisms
☐	☐ Clinical data
☒	☐ Dual use research of concern
☒	☐ Plants

Methods

n/a	Involved in the study
☒	☐ ChIP-seq
☒	☐ Flow cytometry
☒	☐ MRI-based neuroimaging

Clinical data

Policy information about clinical studies

All manuscripts should comply with the ICMJE guidelines for publication of clinical research and a completed CONSORT checklist must be included with all submissions.

Clinical trial registration	This study was not a clinical trial
Study protocol	*Note where the full trial protocol can be accessed OR if not available, explain why.*
Data collection	*Describe the settings and locales of data collection, noting the time periods of recruitment and data collection.*
Outcomes	*Describe how you pre-defined primary and secondary outcome measures and how you assessed these measures.*

Plants

Seed stocks	No plants were included in the study
Novel plant genotypes	*Describe the methods by which all novel plant genotypes were produced. This includes those generated by transgenic approaches, gene editing, chemical/radiation-based mutagenesis and hybridization. For transgenic lines, describe the transformation method, the number of independent lines analyzed and the generation upon which experiments were performed. For gene-edited lines, describe the editor used, the endogenous sequence targeted for editing, the targeting guide RNA sequence (if applicable) and how the editor was applied.*
Authentication	*Describe any authentication procedures for each seed stock used or novel genotype generated. Describe any experiments used to assess the effect of a mutation and, where applicable, how potential secondary effects (e.g. second site T-DNA insertions, mosiacism, off-target gene editing) were examined.*
